# On Treatment of Rheumatism in New York Hospital

**Published:** 1854-09

**Authors:** J. B. Chapin

**Affiliations:** Resident Physician


From the N. Y. Med. Times.

*On Treatment of Rheumatism in New York Hospital* By J. B. Chapin, M.D., Resident Physician.

The plan of treatment usually pursued was: If the patient presented himself with unusual excitement of the skin and pulse, to administer a mixture of sulphate of magnesia and tartarized antimony until the skin was relaxed, and the pulse reduced to a more natural standard. The Rochelle salt was then directed in drachm doses, every two or three hours during the day time, till the urine was rendered akaline, when it was gradually suspended. A lotion of carb. potass. ℥j. with opium ʒij. to the pint of water, was not attended with disagreeable consequences, with the exception occasionally of some ulceration about the fauces,— in no case was its action so severe upon the bowels as to require its entire suspension. The persons attacked were in the full vigor of health, and the character of the disease acute in its form. The frequency of its administration of the remedy was governed very much by the reaction of the urine.

On the admission of the patient, the urine was tested; and, in all cases, was found to be of acid reaction, and the secretion of the skin presented the usual acid odor. The treatment was generally commenced the second or third day after admission, and the urine was rendered of decided alkaline reaction in an average of five days after its commencement; the longest period it resisted the alkaline reaction having been twenty days, and the shortest two. The secretions of the skin have not, I believe, been noticed to alter. In one case, attended with profuse perspiration, which yielded readily to treatment, the colored shirt the patient wore entirely lost its color; and it was suggested whether the same change did not take place in the perspiration as in the urine. The average amount of the salt administered was from five to seven ounces.

The average date of commencing improvement was seven days after commencement of treatment, coinciding, in the large majority of the cases, with the commencing alkalinity of the urine. The improvement was invariably permanent, and after the urine was rendered alkaline, *no new articulations were affected*, as a general rule.

The average period of convalescence was twelve days after admission, and the whole duration of the disease, including the period previous to admission, was twenty-two days. Of thirty cases treated by Dr. Swett, during April and May, 1853, during which time no uniform course of treatment was pursued, the average duration was five and a half weeks. One of the most gratifying results of the alkaline treatment was the diminished frequency of cardiac complications. Twenty-one of the twenty-five were free from any complication, three were *admitted* with arotic obstruction, and one with regurgitation. Not one patient was attacked with any heart complication during the treatment of the disease. Comparing this result with the practice last year, it was found that four had mitral regurgitation, six arotic complication, and three suffered from pericarditis; thirteen in all, out of thirty.

